# Supplementary material for: Insights into protein post-translational modification landscapes of individual human cells by trapped ion mobility time-of-flight mass spectrometry
Source: Nat Commun. 2022 Nov 25;13:7246. doi: 10.1038/s41467-022-34919-w (PMC9700839; doi:10.1038/s41467-022-34919-w)
Supplement: Supplementary file 1 — Supplementary Information [file 41467_2022_34919_MOESM1_ESM.pdf]

Supplementary Information for Orsburn et al., 2022

Search tool	Directly compatible	pasefRiQ reporter ions	Utilizes ion mobility	Unbiased PTM discover	Reporter ion run matching	Software type
MaxQuant	Yes	Yes	Yes	No	Yes	Closed source freeware
MSFragger	Yes	Through commercial PD node	Yes, but not through PD nodes	Yes	No	Open source
MetaMorpheus	No	No	No	Yes	No	Open source
Bolt	Yes	No	Yes	Yes	No	Commercial
PEAKS	Yes	Yes	Yes	No	No	Commercial
Sequest	No	Through commercial PD node	No	No	No	Commercial
MSAmanda	No	Through commercial PD node	No	No	No	Open source

Supplementary Table 1. A summary of proteomics tools and their relative compatibility with TIMSTOF data files.

Instrument settings	
LCMS	EasyNLC1200
Column	IonOpticks Aurora 25cm
Trap	PepMap100 C-18 3cm 75µm
Flow rate	200nL/min
Buffers	0.1% formic acid, 80% acetonitrile 0.1% formic acid
Source	Bruker Daltronic CaptiveSpray
Column temperature	50C
ESI voltage	1650V
Number of Ramps	10 ramps, unless specifically noted
Cycle time	2.2 seconds for 10 ramp files, 1.1 s for 5 ramp files
Quadrupole Isolation Width	Default 2 Da for ions < 700, 3 Da for ions > 700
Optimized Isolation Width	1.5 Da for all masses
TIMS ramp, default	0.6 - 1.6
Optimized TIMS ramp TMT peptide IDs	0.8 - 1.3
Optimized TIMS ramp TMT protein IDs	0.7 - 1.5
TIMS Step 1 Fragmentation energy	75ev
TIMS Step 2 Fragmentation energy	25ev

Supplementary Table 2. A summary of the LCMS method and parameters used in this study.

Supplemental Figures

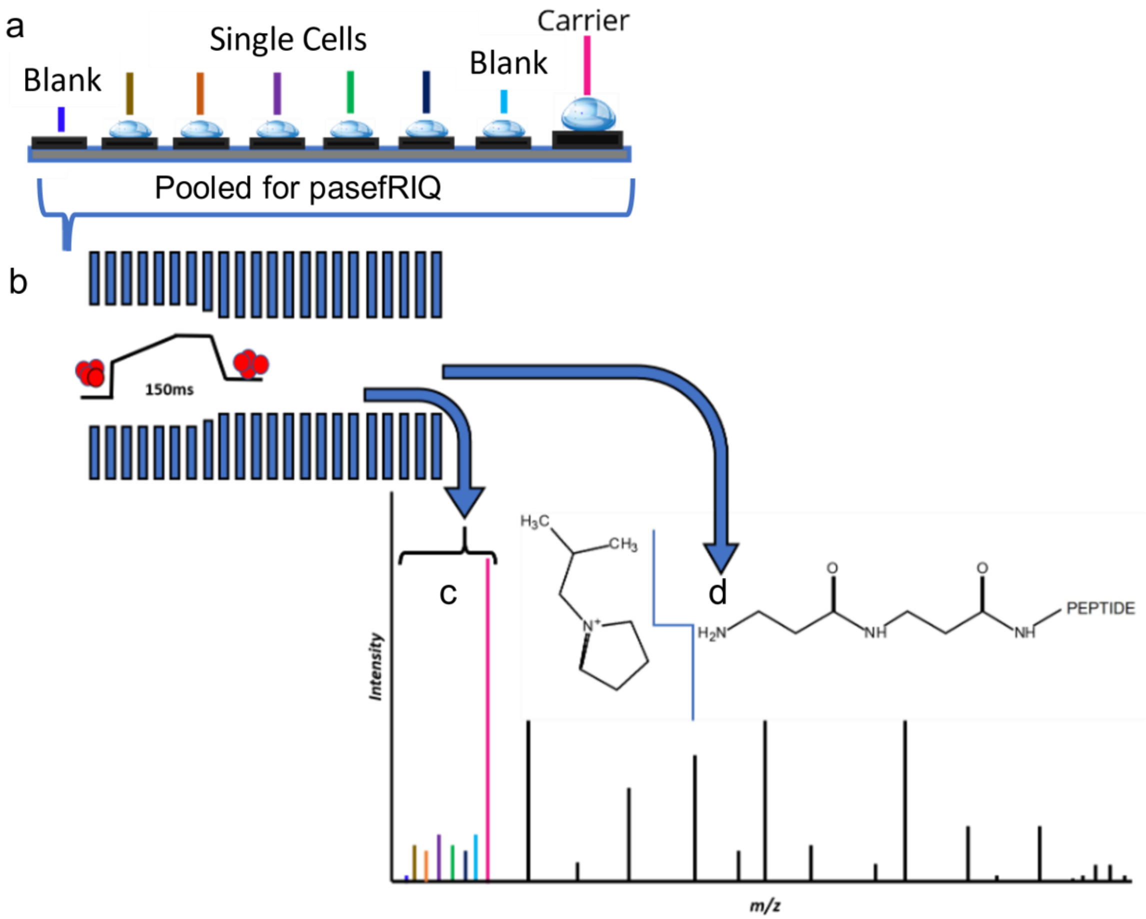

Supplementary Figure 1. A cartoon illustrating the general strategy of analyzing single cells with pasefRiQ. A. Single cells and method blank control containing sorting buffer, but not a cell are sorted into wells of a receiving plate. Digested and labeled peptides are pooled for analysis. B. LCMS with a two stage pasef ramp is performed with ion accumulation occurring over a ramp time of 150 milliseconds. C. The resulting peptide is fragmented once using a high collision energy and pre-pulse trapping parameters optimized to capture low mass fragment ions for quantification. D. A second fragmentation even with lower collision energy and conditions to capture higher m/z fragment ions provide peptide sequencing information. The spectra from the two scans are combined in real time by the mass analyzer hardware.

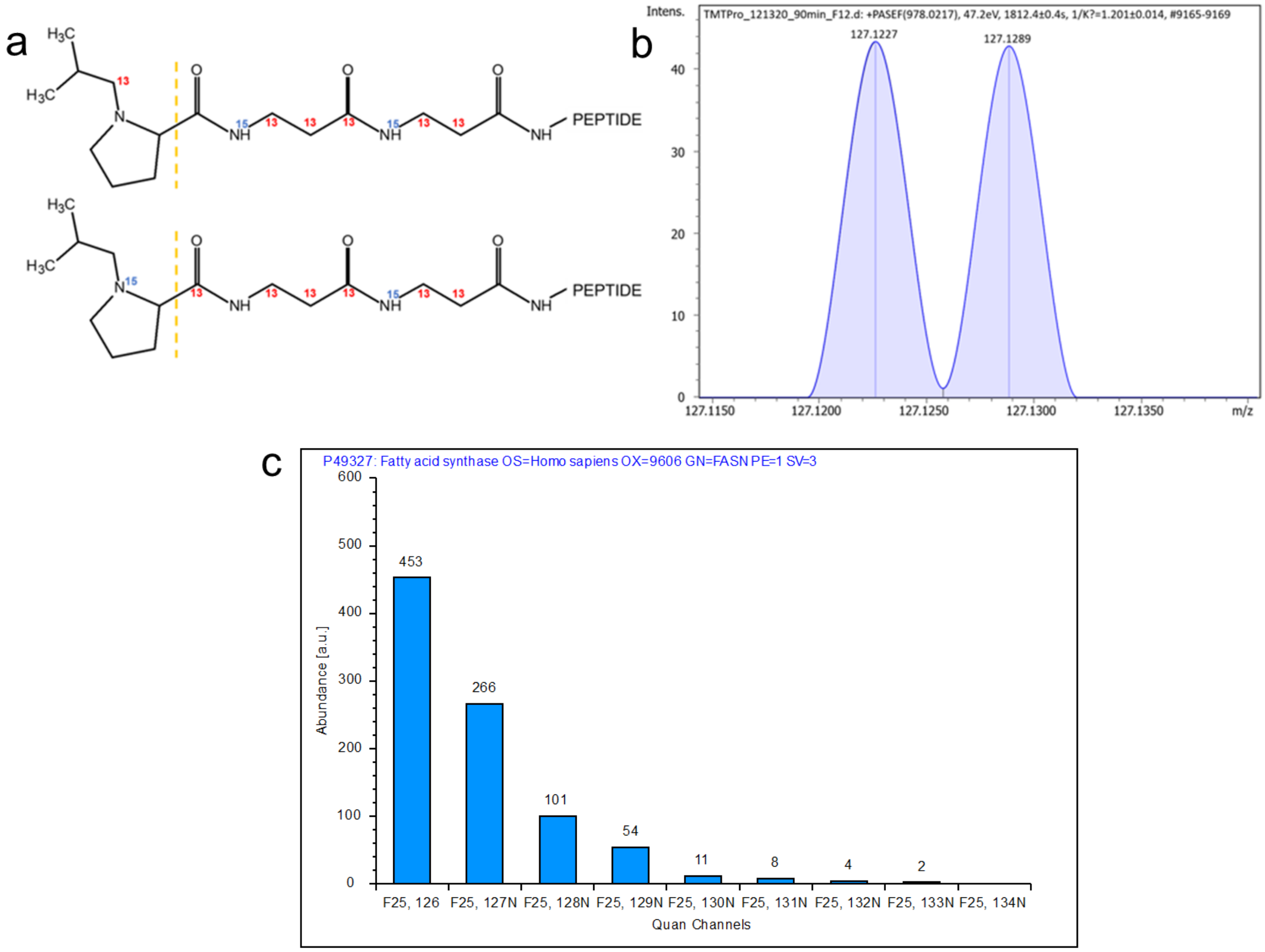

Supplementary Figure 2. Overview of the TMTPro dilution series. A. The structure of the 127N (top) and 127C (bottom) reporter ions B. A representative image from the liberated reporter ion regions of a labeled peptide digest demonstrating that near-baseline separation of these ions can be achieved under some conditions. C. A representative barplot of the summed intensity values for a protein from this dilution series.

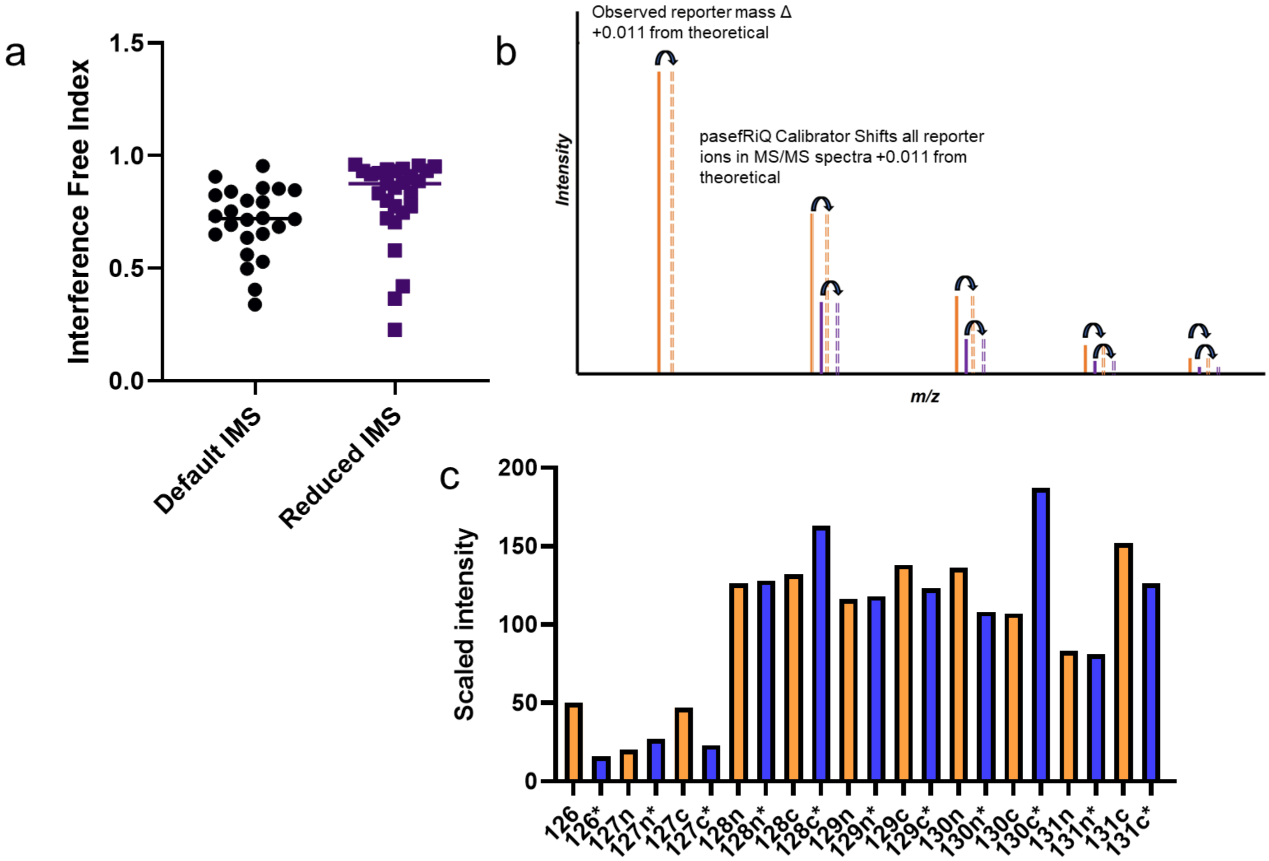

Supplementary Figure 3. Results of optimization to reduce coisolation interference in pasefRiQ data. A. The interference free index of all Met6 peptides for two 60 minute acquisition experiments comparing the isolation interference of the default ion mobility isolation window to a reduced and optimized ion mobility ramp window. The line defines the median value of n=24 peptides for the three Default IMS experiments and 27 peptides n=27 peptides for the 3 Reduced IMS experiments. B. An illustration describing the MS/MS recalibration of reporter ions with the pasefRiQ Calibrator**.** C**.** The scaled intensity of the Met6 protein in the TMT-TKO standard where * denotes the values following one point recalibration. Source data are provided.

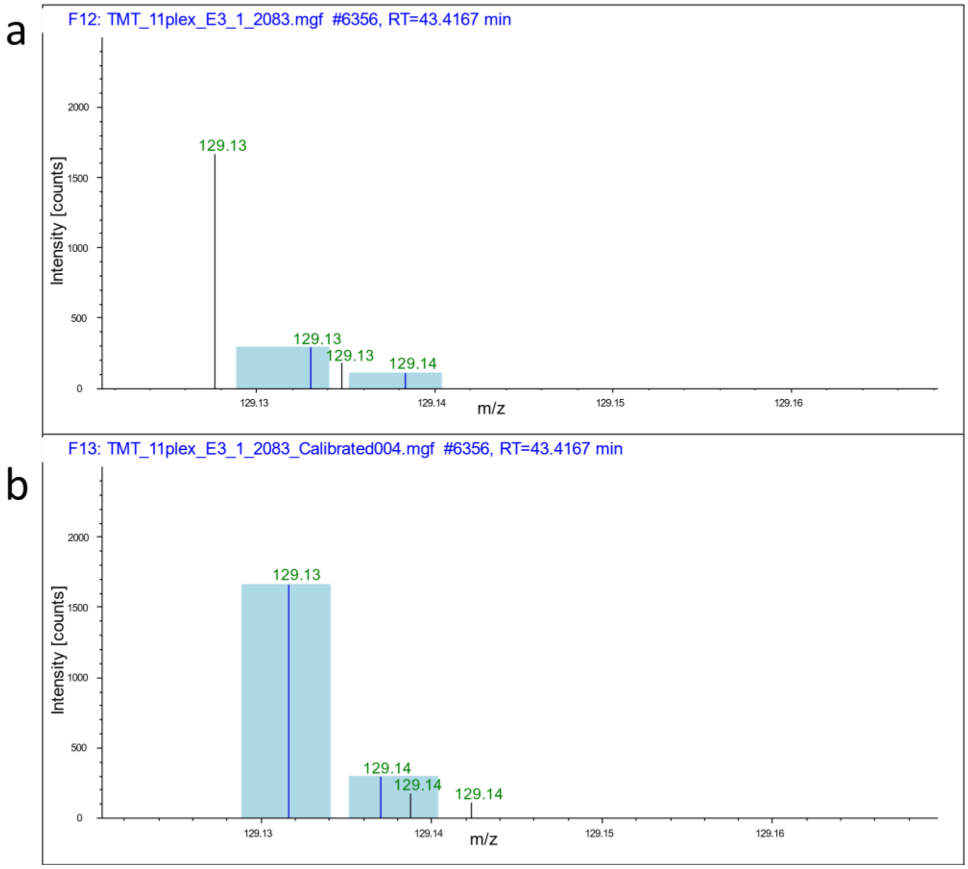

Supplementary Figure 4. An example of the reporter ions integrated for quantification prior to and post recalibration. The blue shaded window indicates the mass range where the reporter ion was sought for detection. An ion fragment in black was not used for quantification and blue was the ion used for quantification. A. Example MET6 peptide AYTYFGEQSNLPK reporter ion 129n and 129c with 20 ppm integration demonstrating failed integration of the major ion peak. B. The same peptide following recalibration demonstrates the correct incorporation of the high abundance reporter ion peak.

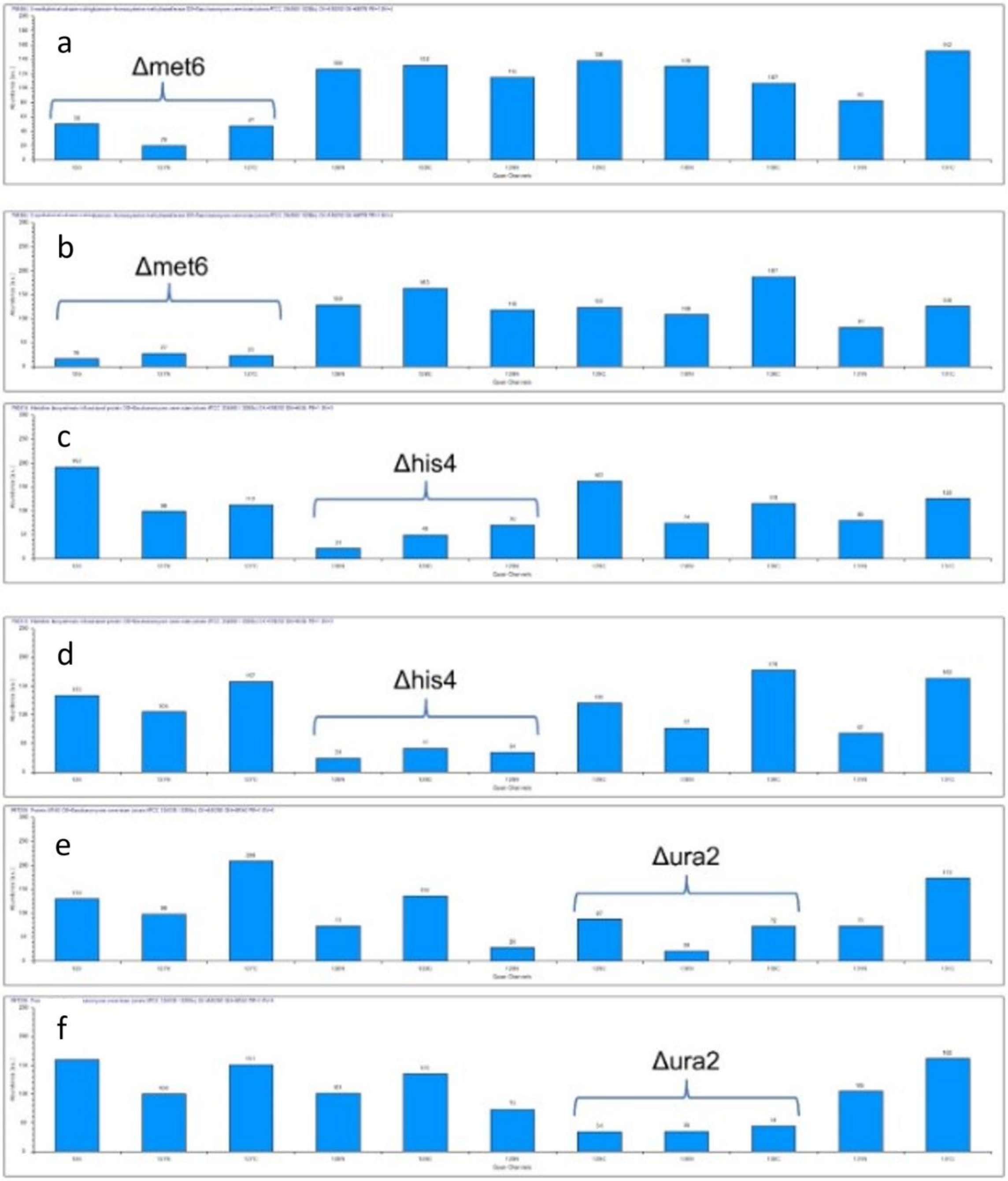

Supplementary Figure 5. Comparison of a triple knockout standard prior to and following recalibration A.,C.,E. The scaled intensities of the Met6, His4, and Ura2 proteins from the TMT-TKO standard using an optimized pasefRiQ workflow integrated with a 20ppm mass tolerance window. B.,D.,F. The scaled intensities of the same respective files following a 0.004 Da recalibration with the *pasefRiQCalibrator*.

a

b

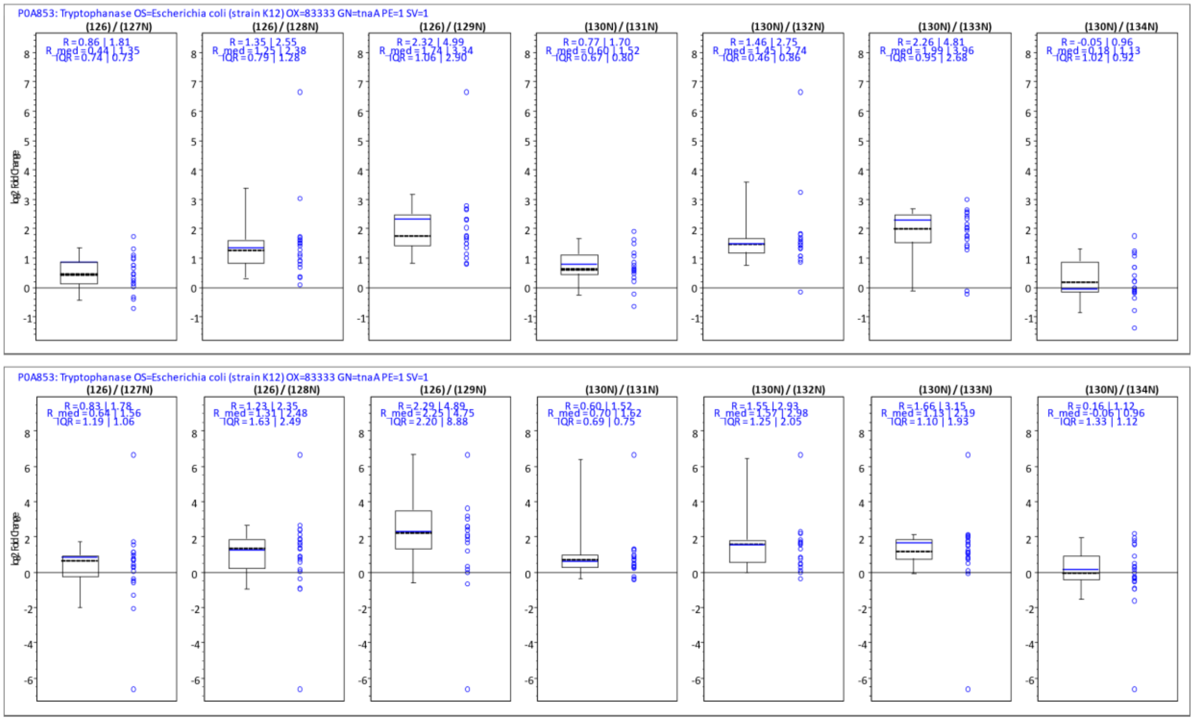

Supplementary Figure 6. Boxplots demonstrating the raw peptide intensities for two of the carrier proteome samples summarized in Figure 2D. A. The peptide intensity values for the lowest relative carrier load of 30x more than any other sample. B. The same for the highest relative carrier load approximating 180 times that of any other sample. The solid line represents the R(log)2. The dotted line displays the median ratio R(log2). The box represents the interquartile range between the 25th and 75th quartiles. Values are derived from n =3 independent experiments. Source data are provided.

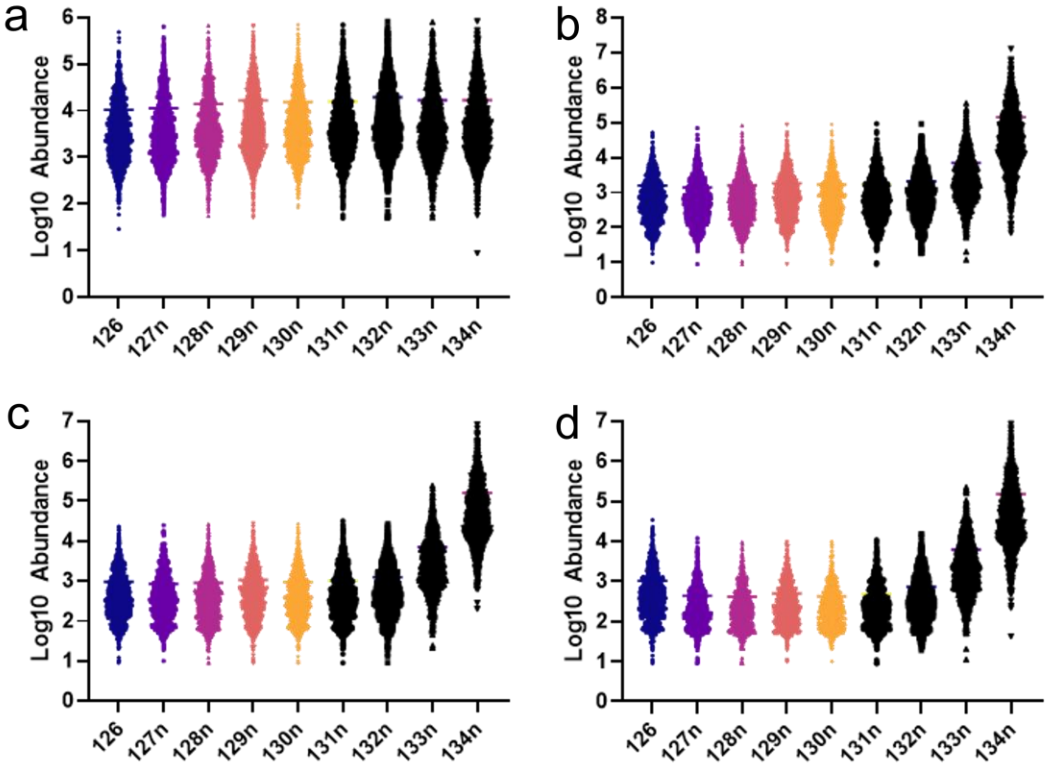

Supplementary Figure 7 Scatter plots demonstrating the increasing effects of ratio distortion observed with increasing carrier channel loads: A. 1x carrier B. 50x carrier. C. 100x carrier. D. 500x carrier. In all figures the box represents the 25th to 75th quartiles with a line denoting the median.. All values are derived from n=3 independent experiments with individual protein values used as each datapoint. Source data are provided.

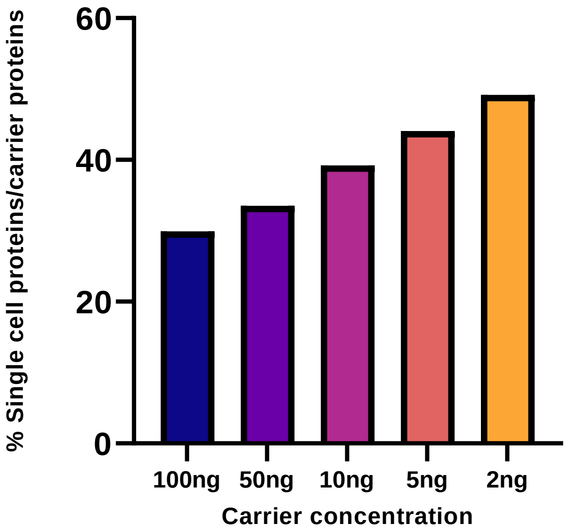

Supplementary Figure 8. Lower concentrations of carrier corresponded to increasing signal from each protein per cell when compared to the identifications from the carrier channel. Source data are provided.

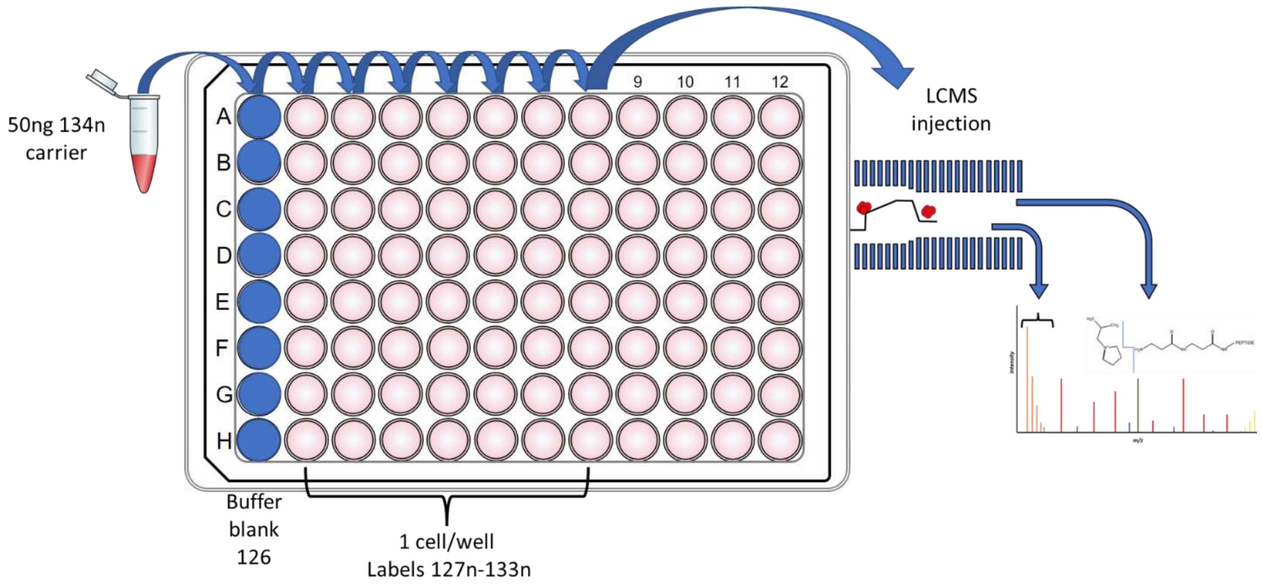

Supplementary Figure 9. An illustration of the sample workflow for pasefRiQ analysis of single H358 cancer cells

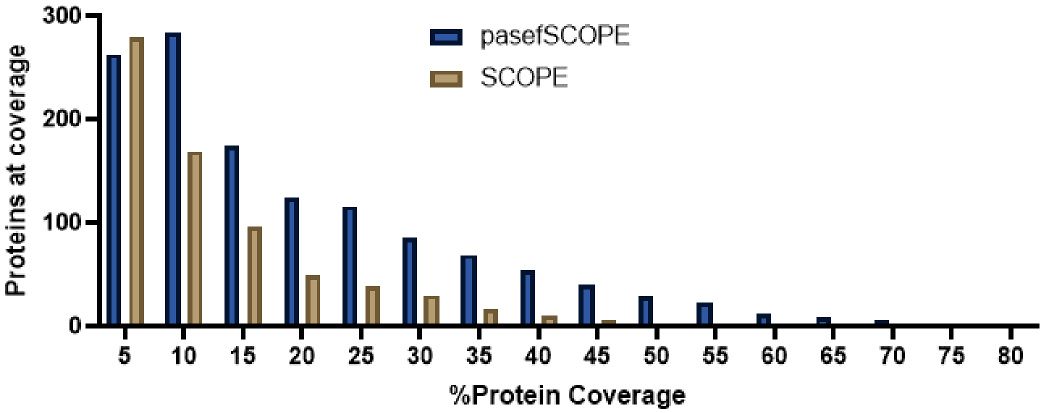

Supplementary Figure 10. A plot comparing the relative percent sequence coverage of each protein identified in a pasefRiQ experiment compared to published SCOPE2 data using a D30 Orbitrap system. Source data are provided.

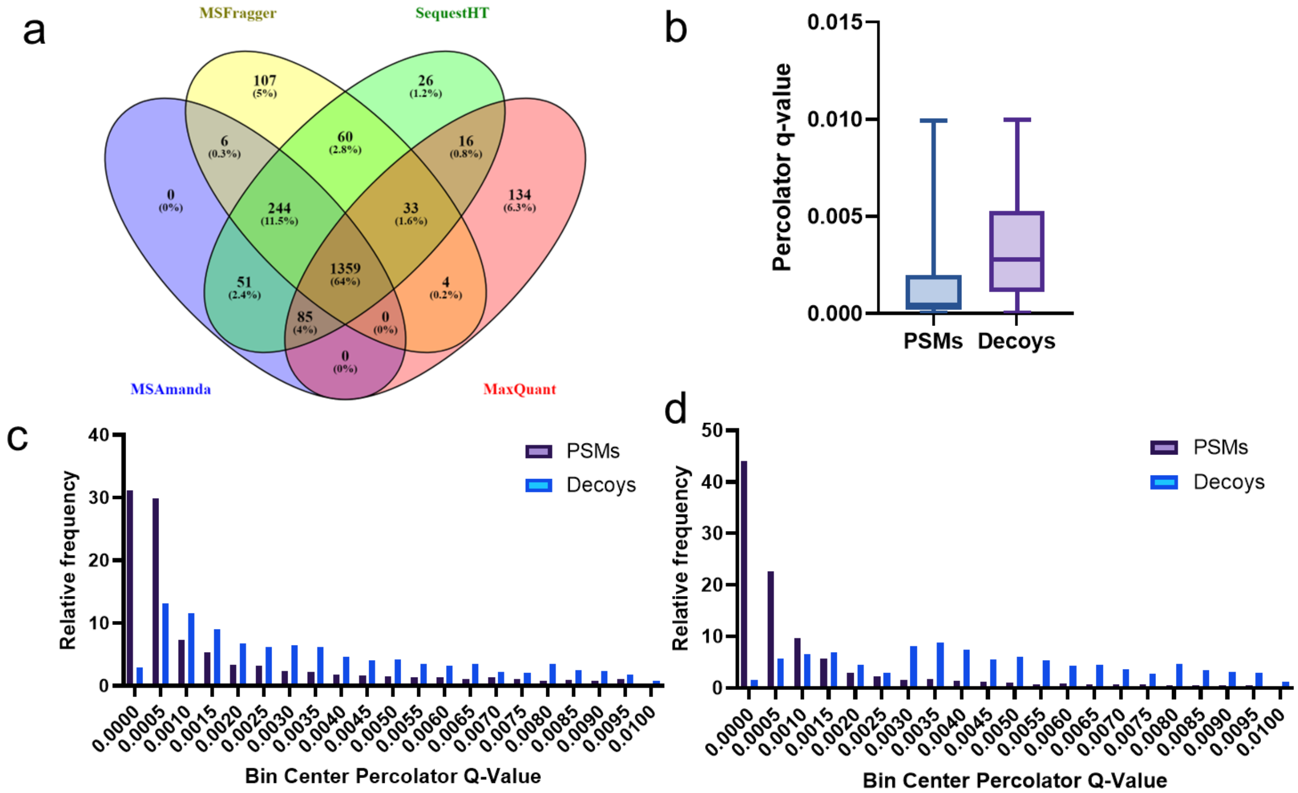

Supplementary Figure 11. Characteristics of peptide spectral matches from single human cells. A. A comparison of the protein level identifications obtained by using four well-characterized search engines to process pasefRiQ H358 single cell data. B. The Percolator q-value distributions of peptide spectral matches with an observed PTM in single cells compared to the decoy peptide q-value distribution for the PTM analysis workflow. In this figure, the box represents the 25th to 75th quartiles with a line denoting the median. The whiskers extend from min to max C. A frequency distribution of the q-values of peptide spectral matches with PTMs compared to all decoy peptide spectral match q-value scores. C. A histogram plotting the relative frequency of percolator q-values for modified PSMs vs decoy matches in a decoy search. D. The same analysis as C using these same data with no PTMs included in the search parameters. Source data are provided.

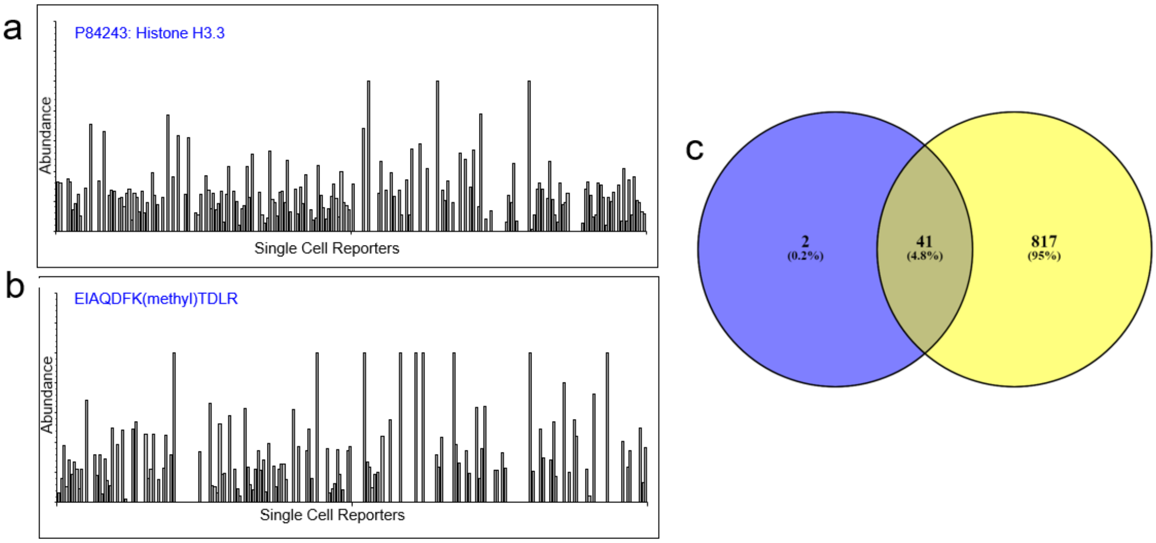

Supplementary Figure 12. The relative abundance of histone proteins and PTMs across all single cells analyzed in this study. A. A bar plot demonstrating the identification rate and relative abundance of Histone 3.3 across 230 single H358 cells. B. A bar plot demonstrate the relative intensity of an identified methylation site on Histone 3.3. C. A Venn diagram representing the number of phosphorylation sites observed in single human cells compared to those observed in a bulk cell lysate analysis by using a single search tool.

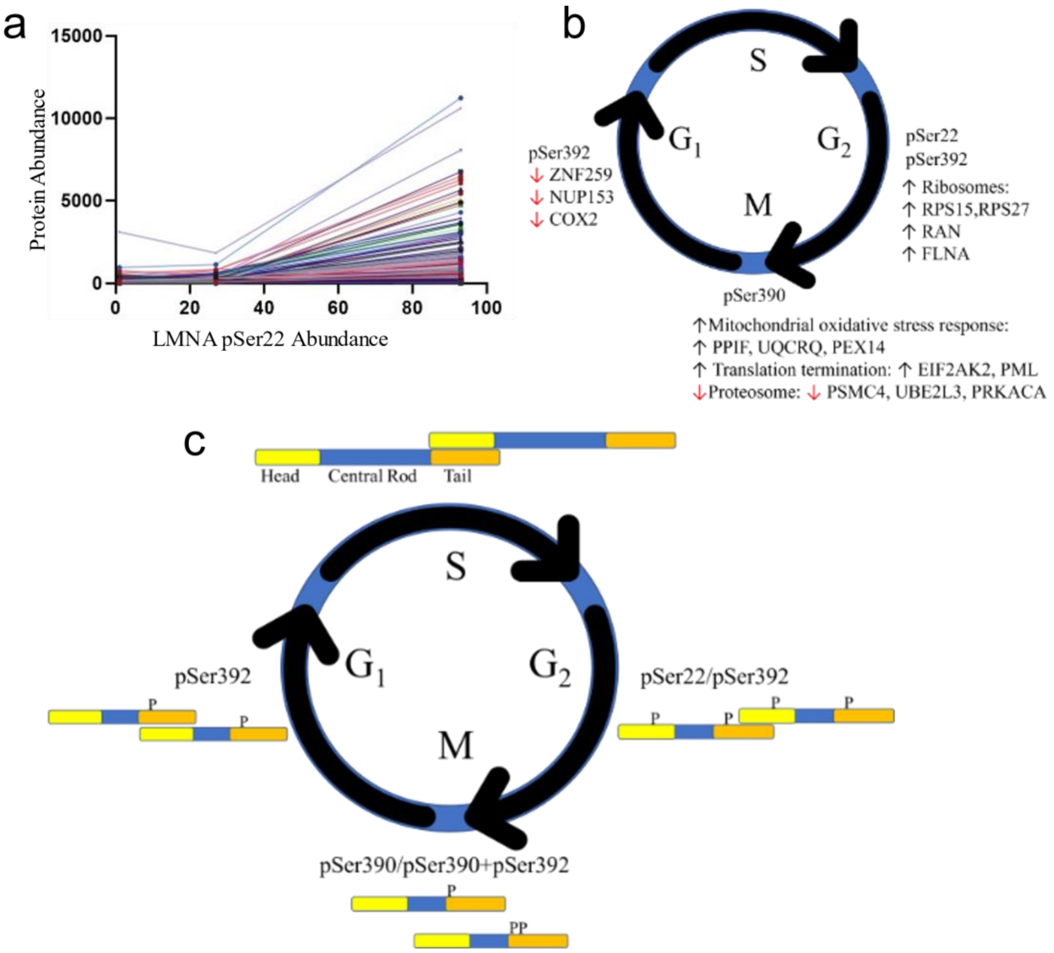

Supplementary Figure 13. Correlation of LMNA phosphopeptides with other mitotic markers. A. Example correlation plots between LMNA Ser22 phosphopeptide abundance and proteins identified as cell cycle dependent by gene ontology analysis. B. A summary of peptides correlating with three LMNA phosphopeptides. C. A proposed mechanism of the activity of each phosphopeptide through the cell cycle of H358 cells. Source data are provided.

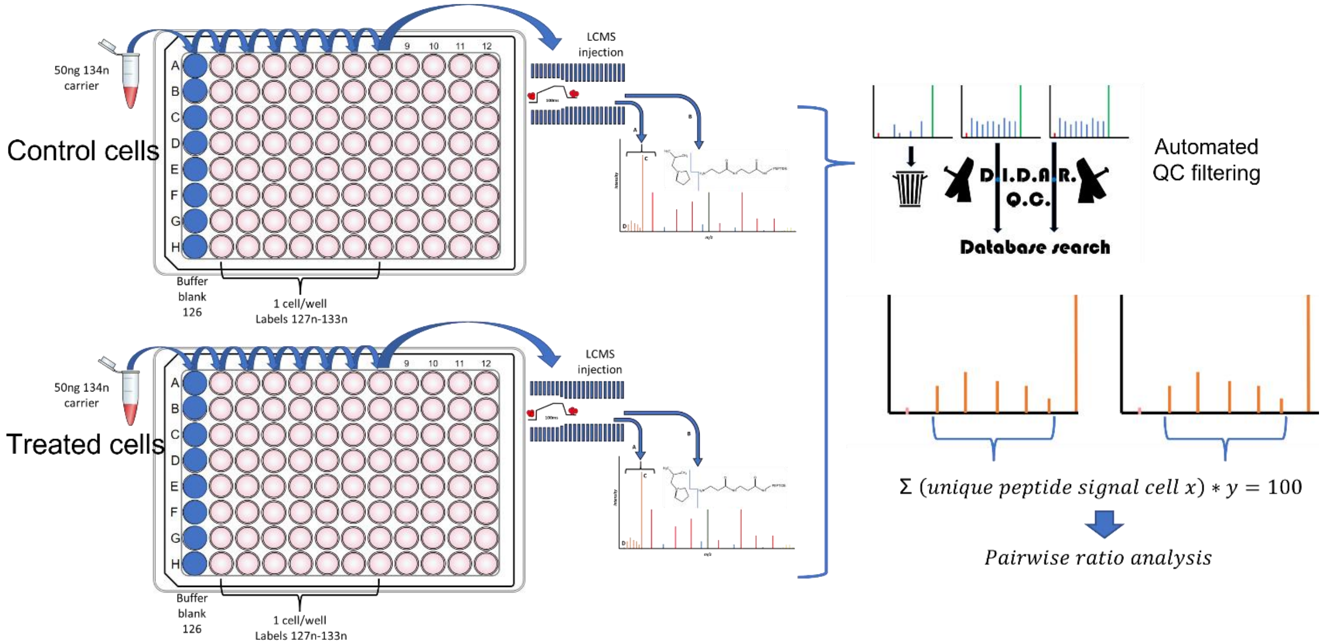

Supplementary Figure 14. A cartoon demonstrating the overall experimental design, quality control and data analysis pipeline for the comparative analysis of 276 single cells in a drug treatment analysis.

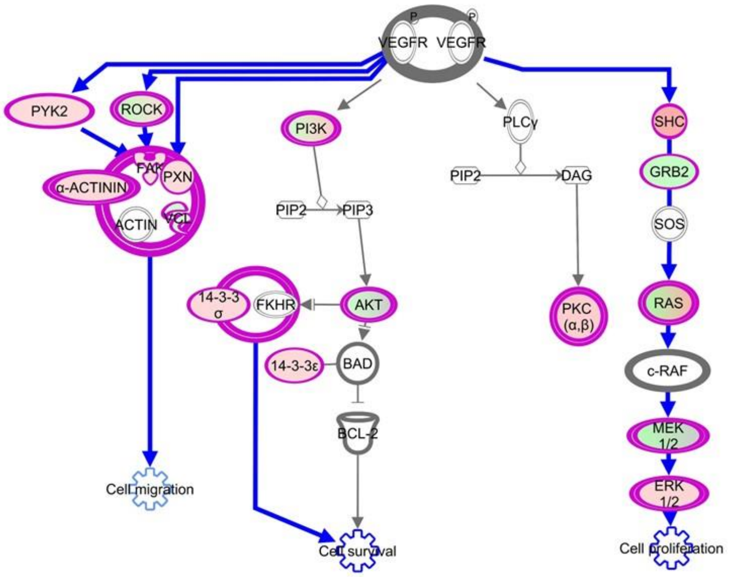

© 2000-2021 QIAGEN. All rights reserved.

Supplementary Figure 15. Pathway analysis indicated the VEGF pathway is the single most altered pathway in H358 cells following 40 hours of sotorasib treatment in single cells.

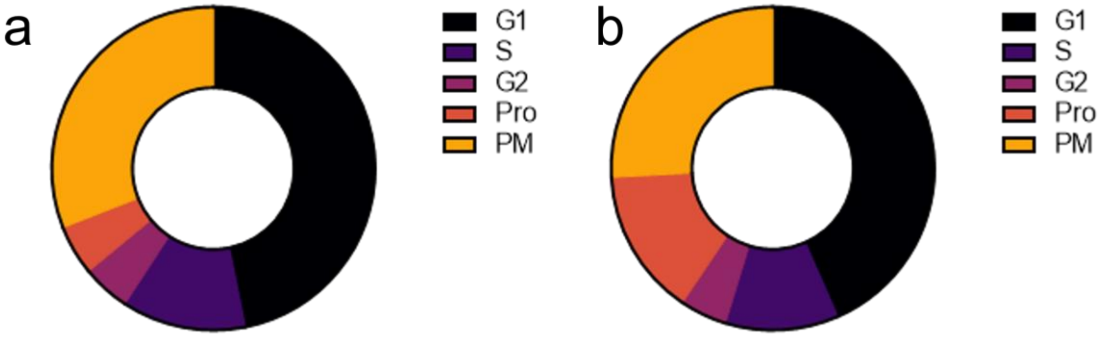

Supplementary Figure 16. A. Shifts in cell cycle related protein abundance between control H358 cells (A) and Sotorasib treated cells (B). Source data are provided.

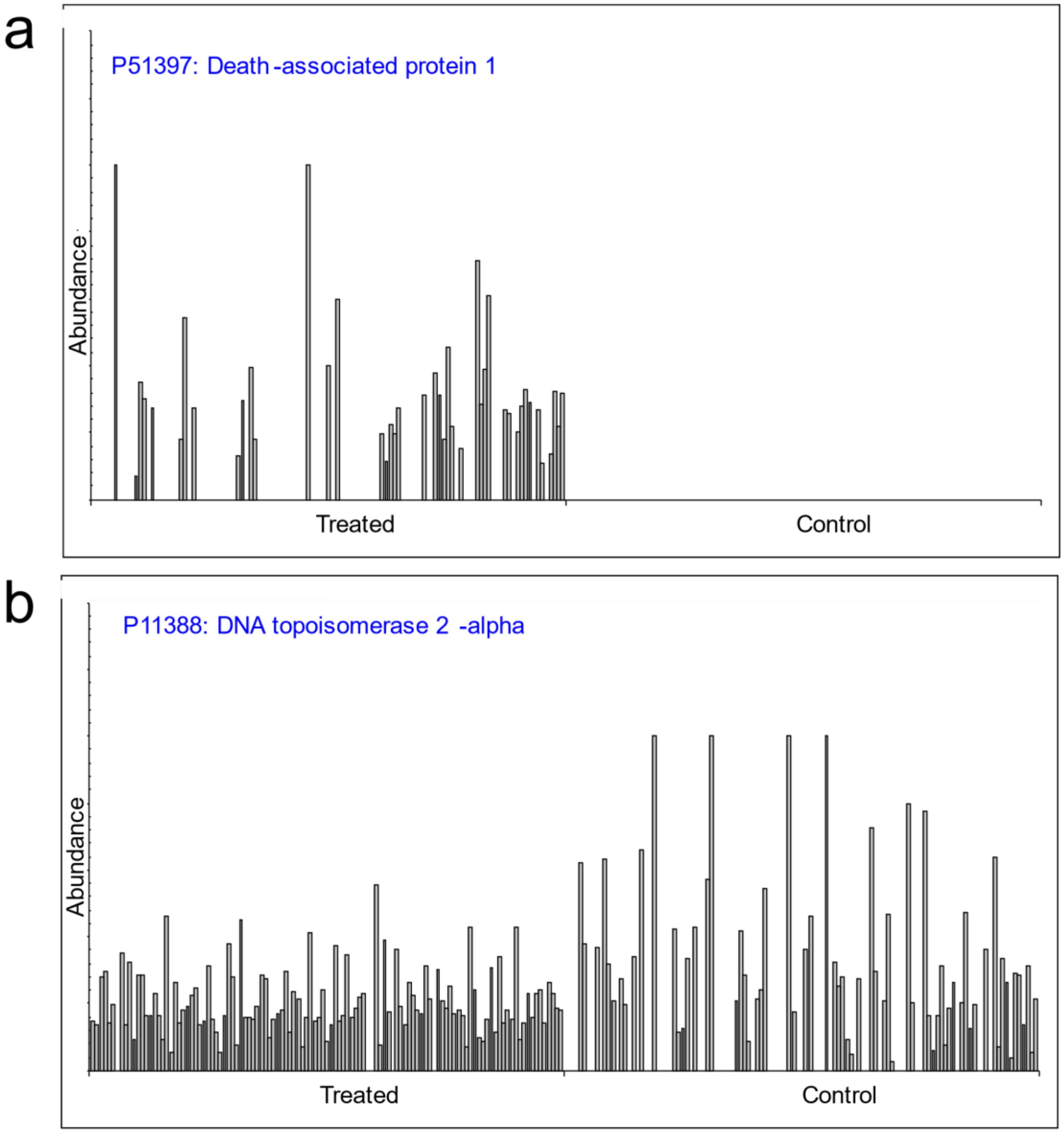

Supplementary Figure 17. A. Visualization of the relative abundance of the DAP1 protein across control and treated cells demonstrating an observation that may be driven by a relatively small cellular subpopulation. B. The relative abundance of the TOP2A protein across cells demonstrating a mechanism that appears more relatively homogenous following drug treatment.

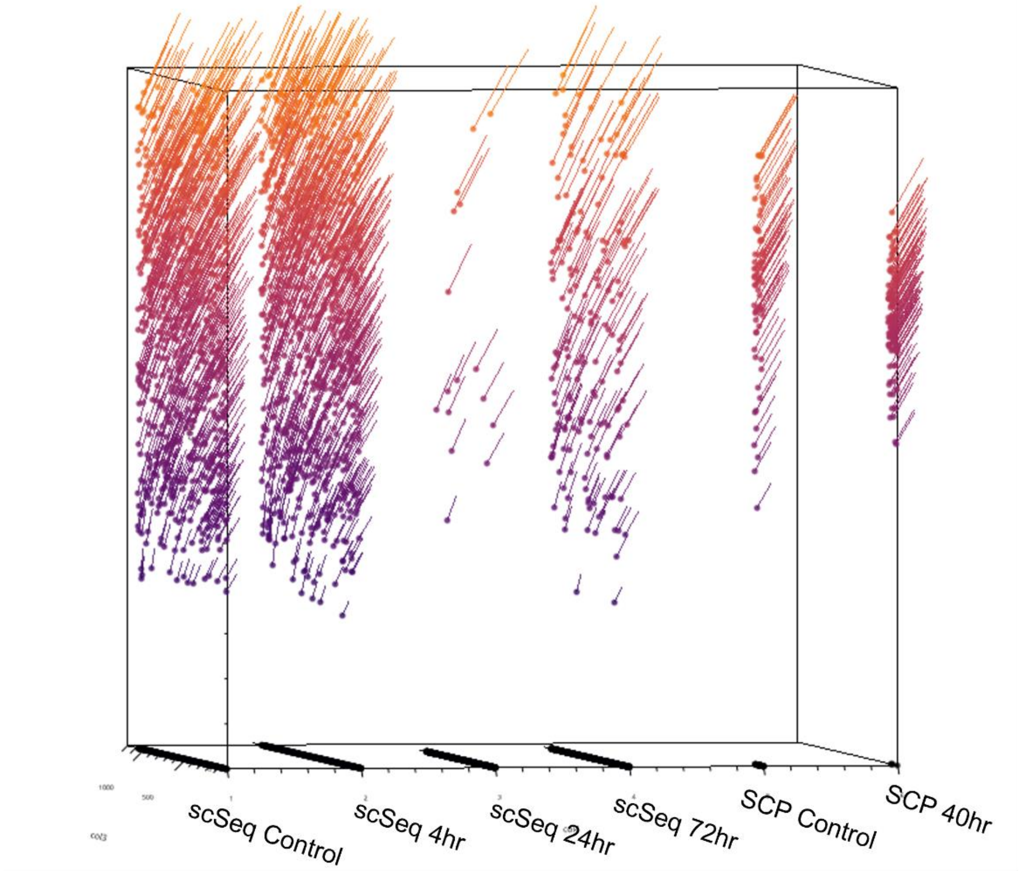

Supplementary Figure 18. A comparison of the transcript and protein expression levels of TOP2A across approximately 4,000 and 230 single cells respectively. Source data are provided.

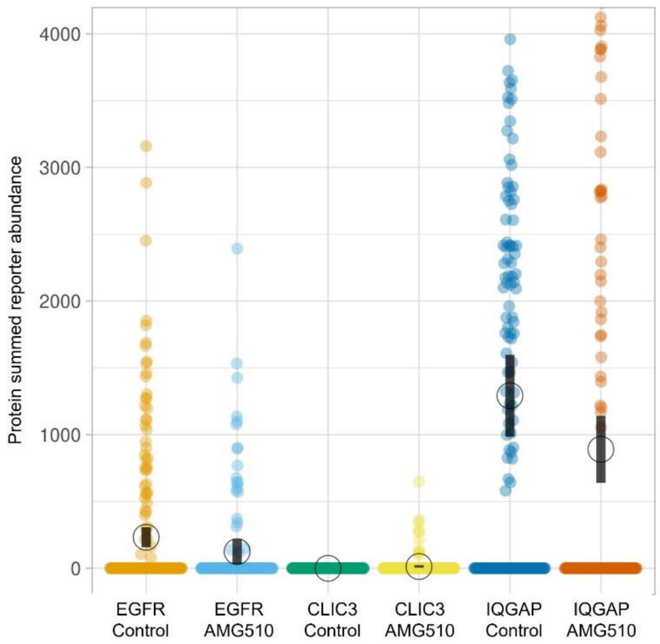

Supplementary Figure 19. A visualization of the abundances of respective proteins across all single cells analyzed. Source data are provided.
