# Supplementary material for: Insights into protein post-translational modification landscapes of individual human cells by trapped ion mobility time-of-flight mass spectrometry
Source: Nat Commun. 2022 Nov 25;13:7246. doi: 10.1038/s41467-022-34919-w (PMC9700839; doi:10.1038/s41467-022-34919-w)
Supplement: Supplementary file 2 — Description of Additional Supplementary Files [file 41467_2022_34919_MOESM2_ESM.docx]

**Description of Additional Supplementary Files**

**Supplementary Data 1.** Data related to Fig. 1A/B and Fig. 2C-2E. A summary of optimization experiments to develop pasefRiQ and all protein and peptide quantification data from a dilution series of K562 spanning a 4-order dilution series.

**Supplementary Data 2.** Data related to Figure 2 and Supplementary Figure 10. A comparison of a pilot analysis of H358 single cells compared to publicly available files from the SCOPE2 study (Sheet1). The dilution series used to test the carrier proteome effect in pasefRiQ (Sheet 2). Summary results of the TNAa protein at each carrier level.

**Supplementary Data 3.** Data related to Figure 3. MS2Go output of all data obtained from single cells in this study using the MSAmanda, MSFragger and Sequest search engines with Percolator quality filtering.

**Supplementary Data 4**. Data related to Supplementary Figure 11. Proteins identified in single cells using 4 separate search engines. Sheet 1 is a composite of results from MSFragger, SequestHT, and MSAmanda 2.0 compiled with Percolator within the Proteome Discoverer 2.4 environment. Sheet 2 is the protein output text report from MaxQuant 1.6.17. The remaining sheets are single LCMS file comparisons using the first and last files of batch 1.

**Supplementary Data 5.** Data related to Figure 3C and Supplementary Figure 16. A summary table of 119 cell cycle proteins and their relative levels of detection and abundance across single cells in this study. Related to Figure 3C.

**Supplementary Data 6.** Data related to Table 1. MS2Go output sheet detailing all proteins, peptides and PSMs where PTMs were detected with MSFragger, MSAmanda and SequestHT using Percolator quality filtering and ptmRS for PTM localization.

**Supplementary Data 7**. Data with various confidence metrics for the PTM identifications made in single cells in this study.

**Supplementary Data 8.** Data related to Fig 4C and 4D. A summary output sheet of proteins with PTMs detailing the localization site and total protein coverage.

**Supplementary Data 9.** Data related to Supplementary Figure 13. Correlation coefficients of phosphopeptide abundance compared to proteins as expressed in single cells in this study.

**Supplementary Data 10.** Panther pathway data in support of Figure 7C. Panther pathway analysis of transcripts from single cell seq analysis of 1,000 control vs 1,000 sotorasib treated cells (Sheet 1) with a 2-fold differential cutoff. The same pathway analysis of 115 control vs 115 treated single cells by SCP (Sheet 2).

**Supplementary Data 11.** STRINGDB pathway analysis comparing sotorasib treated cells exhibiting high CLIC3 expression compared to all other cells.
