# Supplementary material for: Insights into protein post-translational modification landscapes of individual human cells by trapped ion mobility time-of-flight mass spectrometry
Source: Nat Commun. 2022 Nov 25;13:7246. doi: 10.1038/s41467-022-34919-w (PMC9700839; doi:10.1038/s41467-022-34919-w)
Supplement: Supplementary file 4 — Reporting Summary [file 41467_2022_34919_MOESM4_ESM.pdf]

nature research

Corresponding author(s): Benjamin C. Orsburn

Last updated by author(s): Nov 9, 2022

# Reporting Summary

Nature Research wishes to improve the reproducibility of the work that we publish. This form provides structure for consistency and transparency in reporting. For further information on Nature Research policies, see our Editorial Policies and the Editorial Policy Checklist.

## Statistics

For all statistical analyses, confirm that the following items are present in the figure legend, table legend, main text, or Methods section.

| n/a | Confirmed | |
|---|---|---|
| ☐ | ☒ | The exact sample size (*n*) for each experimental group/condition, given as a discrete number and unit of measurement |
| ☐ | ☒ | A statement on whether measurements were taken from distinct samples or whether the same sample was measured repeatedly |
| ☐ | ☒ | The statistical test(s) used AND whether they are one- or two-sided<br>*Only common tests should be described solely by name; describe more complex techniques in the Methods section.* |
| ☐ | ☒ | A description of all covariates tested |
| ☒ | ☐ | A description of any assumptions or corrections, such as tests of normality and adjustment for multiple comparisons |
| ☐ | ☒ | A full description of the statistical parameters including central tendency (e.g. means) or other basic estimates (e.g. regression coefficient) AND variation (e.g. standard deviation) or associated estimates of uncertainty (e.g. confidence intervals) |
| ☐ | ☒ | For null hypothesis testing, the test statistic (e.g. *F*, *t*, *r*) with confidence intervals, effect sizes, degrees of freedom and *P* value noted<br>*Give P values as exact values whenever suitable.* |
| ☒ | ☐ | For Bayesian analysis, information on the choice of priors and Markov chain Monte Carlo settings |
| ☒ | ☐ | For hierarchical and complex designs, identification of the appropriate level for tests and full reporting of outcomes |
| ☐ | ☒ | Estimates of effect sizes (e.g. Cohen's *d*, Pearson's *r*), indicating how they were calculated |

*Our web collection on statistics for biologists contains articles on many of the points above.*

## Software and code

Policy information about availability of computer code

| | |
|---|---|
| Data collection | Data was collected using a TIMSTOF Flex mass analyzer operating with TIMS Control 3.0 (Bruker Daltronic). |
| Data analysis | Bruker instrument output data was converted to the universal mascot generic format text files with MSConvert 3.0.2 Developer Build. MGFs were calibrated when applicable with the pasefRiQCalibrator which was developed for this work (https://github.com/orsburn/pasefRiQCalibrator(https://github.com/orsburn/pasefRiQCalibrator). Quality control analysis and filtering was performed with DIDARSCPQC (https://github.com/orsburn/DIDARSCPQC).The output MGFs were searched with Proteome Discoverer v2.4 (Thermo Fisher) as noted in the methods section, MSFragger V17, MaxQuant 1.6.17 and MetaMorpheus 320. Downstream analysis was performed with SimpliFi (www.simplifi.protifi.com) and with Ingenuity Pathway Analysis as noted in the text. Cell cycle analysis from single cell proteomic data was performed with: https://github.com/orsburn/SCP_cell_cycle_stripping and the visualization and integration of single cell RNASeq data was performed with https://github.com/orsburn/gluevizSingleCell using GlueViz 1.0.0 in Anaconda Navigator 3.0 |

For manuscripts utilizing custom algorithms or software that are central to the research but not yet described in published literature, software must be made available to editors and reviewers. We strongly encourage code deposition in a community repository (e.g. GitHub). See the Nature Research guidelines for submitting code & software for further information.

## Data

Policy information about availability of data

All manuscripts must include a data availability statement. This statement should provide the following information, where applicable:

- Accession codes, unique identifiers, or web links for publicly available datasets
- A list of figures that have associated raw data
- A description of any restrictions on data availability

All instrument vendor files, quality control filtered peak files and output reports are available through the MASSIVE public repository (www.massive.ucsd.edu) as

accessions: 88757,88796,88144 and 88157. These have been made publicly available at this update.

# Field-specific reporting

Please select the one below that is the best fit for your research. If you are not sure, read the appropriate sections before making your selection.

- [x] Life sciences
- [ ] Behavioural & social sciences
- [ ] Ecological, evolutionary & environmental sciences

For a reference copy of the document with all sections, see nature.com/documents/nr-reporting-summary-flat.pdf

# Life sciences study design

All studies must disclose on these points even when the disclosure is negative.

| | |
|---|---|
| Sample size | A total of 441 single human NCI-H-358 cells were analyzed in this study. This number of samples was used simply because that was the maximum instrument time available for this study. We believe this sample size is sufficient for this proof of concept study due to recent work by Sam Payne at BYU which estimated the number of single cell proteomes necessary to gain insight into an effect. (DOI: 10.1016/j.mcpro.2021.100085) |
| Data exclusions | All cells labeled with the 133n reagent were excluded from analysis due to apparent signal inflation due to impurities of the commercial tagging reagent 134n. Spectra were removed from downstream analysis by the DIDAR QC tool if no clear reporter signal from a single cell was observed within a 0.005 Da mass tolerance window for reporter ion regions utilized for single cells. In addition, four LCMS runs were flagged by DIDAR as containing extensive signal in the 126 blank channel, suggesting contamination with the individual plate, which was removed from downstream consideration. |
| Replication | Including control, method blank and excluded 133n cells, each LCMS injection measured 6 multiplexed human cells. 63 replicate experiments are described in this study. Replicates appear to have been successful within the boundaries of this study. |
| Randomization | Single cells were sorted into each open well of a 96 well plate in a random fashion |
| Blinding | Investigators were not blinded during the development of this method due simply to the practicality of one operator with the ability to load one 96 well plate into the instrument at at time. |

# Reporting for specific materials, systems and methods

We require information from authors about some types of materials, experimental systems and methods used in many studies. Here, indicate whether each material, system or method listed is relevant to your study. If you are not sure if a list item applies to your research, read the appropriate section before selecting a response.

## Materials & experimental systems

| n/a | Involved in the study | |
|---|---|---|
| ☒ | ☐ | Antibodies |
| ☐ | ☒ | Eukaryotic cell lines |
| ☒ | ☐ | Palaeontology and archaeology |
| ☒ | ☐ | Animals and other organisms |
| ☒ | ☐ | Human research participants |
| ☒ | ☐ | Clinical data |
| ☒ | ☐ | Dual use research of concern |

## Methods

| n/a | Involved in the study | |
|---|---|---|
| ☒ | ☐ | ChIP-seq |
| ☒ | ☐ | Flow cytometry |
| ☒ | ☐ | MRI-based neuroimaging |

## Eukaryotic cell lines

Policy information about cell lines

| | |
|---|---|
| Cell line source(s) | NCI-H-358 ATCC #5807, K562 peptide digest standard V7461 |
| Authentication | Cell lines were directly received from ATCC and authenticated by that organization. |
| Mycoplasma contamination | Cell lines were verified as free of mycoplasma by the provider. |
| Commonly misidentified lines (See ICLAC register) | No commonly misidentified cell lines were used in this study. |
